# Does level of motivation for change impact post-treatment outcomes in the eating disorders? Protocol for a systematic review with quantitative analysis

**DOI:** 10.1186/s40337-017-0147-1

**Published:** 2017-04-27

**Authors:** Jeanne Sansfaçon, Howard Steiger, Lise Gauvin, Émilie Fletcher, Mimi Israël

**Affiliations:** 1 Douglas Mental Health University Institute, Montréal, Québec Canada; 2 Psychiatry Department, McGill University, Montréal, Québec Canada; 3 Psychology Department, McGill University, Montréal, Québec Canada; 4 CHUM Research Centre, Montréal, Québec Canada; 5 Department of Social and Preventive Medicine, Université de Montréal, Montréal, Québec Canada

**Keywords:** Eating disorders, Anorexia nervosa, Bulimia nervosa, Binge-eating disorder, Other specified feeding or eating disorder, Motivation for treatment, Self-determination theory, Transtheoretical stage of change model, Readiness for change, Systematic review, Meta-analysis

## Abstract

**Background:**

Eating Disorders are highly prevalent and widespread mental health problems, with marked risk of chronicity and refractoriness to treatment. Affected individuals are hesitant to change their behaviours and therefore struggle to maintain motivation for therapy. This review aims to produce the first high-quality meta-analysis of the literature on the impact of level of motivation for change on post-treatment outcomes in anorexia nervosa, bulimia nervosa, binge-eating disorder, and other specified feeding or eating disorder (OSFED).

**Methods:**

A systematic review will be conducted using Cochrane library, Embase, MEDLINE, and PsychINFO. Research registrars and bibliographies of included articles will be screened, and experts will be contacted. The search strategy consists of terms related to eating disorders, motivation, and outcome. Randomized controlled trials, clinical controlled trials, time series, and before-after studies will be included. Participants will be adolescents and adults who are diagnosed with anorexia nervosa, bulimia nervosa, binge-eating disorder or OSFED and who are entering psychotherapy treatment. The predictor studied is defined as motivation for change at the beginning of treatment. The primary outcome will be an overall change in eating-disorder symptomatology at the end of treatment and at less than, and over 6-month follow-up. Other outcomes of interest include change in restricting, binging, and compensatory behaviours, change in shape, weight and eating concerns, change in psychiatric comorbidities, weight restoration, and dropout rates. Articles will be selected, data will be extracted, and the risk of bias will be assessed by independent reviewers using forms pre-created on Eppi-Reviewer 4 software. Results will be combined using a random-effects model. Studies of all sizes and qualities will be included in the analyses. Heterogeneity will be examined by funnel plot, Cochran’s Q, and I^2^ statistic. Sensitivity analyses will be performed to account for clinical and methodological differences across studies.

**Discussion:**

This systematic review will help determine the predictive value of motivation for change on treatment outcomes in eating disorders.

**Trial registration:**

Our systematic review protocol is registered with the International Prospective Register of Systematic Reviews (PROSPERO) (CRD42016035285). All modifications will be available on the PROSPERO website, along with the dates, a description, and a justification.

**Electronic supplementary material:**

The online version of this article (doi:10.1186/s40337-017-0147-1) contains supplementary material, which is available to authorized users.

## Background

Eating Disorders (EDs), encompassing Anorexia Nervosa (AN), Bulimia Nervosa (BN), Binge-Eating Disorder (BED), and Other Specified Feeding or Eating disorder (OSFED) are characterized by intense preoccupations with eating, weight and/or body-image, disturbances of eating behaviours leading to altered food ingestion, and impairments in affected individuals’ physical and mental health [1]. A recent epidemiological study indicates lifetime prevalence of AN, BN, and BED to be 0.9, 1.5 and 3.5% in women, respectively [2]. EDs lead to significant suffering, and can become life-threatening. They exhibit among the highest mortality rates of all mental illnesses [3], with mortality rates estimated to 5.1% in AN and to 1.7% in BN, per decade [4–6].

EDs tend to be chronic illnesses and are often perceived as being particularly difficult to treat. Dropout and relapse rates are high. Dropout rate is estimated to be 20–40% in outpatient treatment for AN [7]. Relapse rate post-hospitalization is estimated to be 42% within 1 year of discharge in AN [8] and to be 31% within 2 years of discharge in BN [9]. It has been suggested that poor response to treatment in EDs might be partly attributable to a low motivation to change ED-related habits [10–12]. Indeed, affected individuals are commonly known to be greatly ambivalent towards modifying their behaviours and thus have difficulty maintaining engagement in treatment. It has been shown that lower motivation for recovery predicts dropouts in EDs, and that a high dropout rate correlates with reduced recovery [13–15].

So far, no meta-analytic review has focused on the relationship between motivation for change and treatment outcomes in EDs. A qualitative review published in 2013 examined the effect of motivation at treatment entry on treatment outcome in eating disordered individuals [16]. It reported that, although the effect sizes from individual studies were not combined, pre-treatment motivation seemed to have a medium to large effect on restrictive and binging behaviours, whereas no effect of motivation was found on purging behaviour. In this study, the authors noted issues of heterogeneity in relation to the different measurement tools used to assess motivation, differences in study designs, and type and length of therapy. Many included studies were small and lacked power. A meta-analysis studying general predictors of treatment outcomes in EDs was subsequently published in 2015 [13]. In this study, motivation was one of several predictors studied. It was found that greater motivation to recover was associated with better outcomes at the end of treatment (effect size 0.26) and at follow-up (effect size 0.15), with 9 and 6 studies included in the analysis, respectively. However, this review’s search strategy was not sensitive to identify motivation-related papers and therefore did not capture all relevant articles. The authors partially addressed this limitation by publishing an erratum [17] including additional articles. Their analysis of motivation as a predictor of EDs should nevertheless be interpreted with caution because some more relevant research may have been missed, leading to unreliable results. Finally, three reviews investigated a slightly different issue [18–20]. The authors were interested in whether the use of interventions specifically designed to enhance motivation in EDs translated into increased motivation and improved outcomes. Overall, the reviews reported that motivational interventions increase motivation to change bingeing and reduced actual bingeing behaviour. However, there was little evidence that interventions impacted purging behaviours, restriction, or weight restoration. None of the reviews involved conducting a meta-analysis.

The objective of the present study is to assess the predictive effect of motivation for change upon symptom evolution and dropout rates in AN, BN, BED, and OSFED at end of treatment, as well as at less than, and over 6 months after end of treatment. The primary treatment outcome examined will be overall ED symptomatology. Secondary treatment outcomes assessed will include ED-related concerns (restraint, shape, eating, and weight concerns), binging behaviour, compensatory behaviour (encompassing vomiting, exercise and medication misuse), changes in weight and in psychiatric comorbidities severity as well as dropout rates. Given that narrative reviews of the published research point to an overall positive effect of motivation on treatment outcome, we hypothesize that above and beyond the effects of different treatment modalities for EDs, higher motivation for change will be associated with more favourable treatment outcomes on each of the indices mentioned above, as well as lower dropout rates.

This meta-analysis will be the first to focus on the relationship between motivation for change and post-treatment outcomes in AN, BN, BED, and OSFED. It will be of high quality, with a broad and systematic search strategy as well as a solid methodology following the PRISMA guidelines (see Additional file 1). This methodology will provide a clearer understanding of the predictive effects of motivation on the evolution of overall and individual symptoms pertaining to ED-pathology. Through heterogeneity analyses, this meta-analysis will hopefully lead to new hypotheses regarding why motivation for change may have a greater association with some symptoms over others. Since studies of motivational therapeutic techniques have shown inconsistent results in EDs, developing an empirically-founded understanding of the association between motivation and discreet eating-disorder symptoms would help identify where existing therapies failed, and would be useful to the development of novel motivational techniques specifically suited for EDs.

## Methods

### Eligibility criteria

Studies will be selected according to the criteria outlined below.

#### Study designs

ED treatment studies assessing motivation for change at treatment entry and ED symptomatology at the end of treatment will be included. Studies including randomized controlled trials (RCTs), cluster randomised trials (CRT), and non-randomised clinical controlled trials (non-rCCTs) will be included as well as before-after studies and time series analyses. Given their obvious limitations, observational studies, case reports, case series, and cross-sectional studies will be excluded.

#### Participants

We will review studies involving adolescent or adult participants (>9 years old) who are diagnosed with AN, BN, BED, or OSFED upon entry to treatment. The diagnoses of AN, BN, BED, and OSFED will be based upon established criteria, such as those of the Diagnostic and Statistical Manual of Mental Disorders (DSM) or of the International Classification of Diseases (ICD). To address the changes in nomenclature and diagnostic criteria between DSM-IV and -5, people who were diagnosed as having EDNOS as per DSM-IV criteria will be included in the OSFED category, while subjects diagnosed with AN and BN according to DSM-IV will be coded as AN and BN, respectively. In some instances, trials published before the publication of the DSM-5 describe their participants as having a subthreshold AN or BN, and define “subthreshold” as being participants who binge or purge at least once a week, and otherwise meet AN or BN diagnostic criteria. When this occurs, participants will be included in the corresponding AN or BN category as per DSM-5 criteria. This inevitable recoding procedure will introduce error into borderline variants of EDs, but will retain a valid distinction between presence or absence of clear-cut clinical cases.

#### Intervention

Studies using any kind of psychotherapeutic treatment will be accepted. Interventions included will consist of, but will not be limited to, family-based therapy (FBT), cognitive behavioural therapy (CBT), motivational interviewing (MI), motivational enhancement therapy (MET), dialectic behavioural therapy (DBT), cognitive remediation therapy (CRT), eye movement desensitization and reprocessing (EMDR), mindfulness, psychoeducation, and multimodal strategies. Individual and group therapies as well as self-help app-based and web-based interventions will be included. Self-help manual-based treatments will also be included if they employed published self-help books for the treatment of EDs that are recognized and recommended by experts in the field (eg. “Overcoming binge-eating”, “The overcoming bulimia workbook: Your comprehensive, step-by-step guide to recovery”, or “Getting better bit(e) by bit(e): A survival kit for sufferers of bulimia nervosa and binge-eating disorders”) [21–23]. Other types of treatments, such as pharmacotherapy or alternative treatments like yoga or acupuncture will be excluded.

#### Comparator

Studies with or without a comparison group will be included. If a comparison arm is present, a variety of control interventions will be found. We anticipate that some authors will use waiting-list participants as controls while others will offer them solely reading materials or will have them take part in a different and maybe shorter segment of therapy (for instance, psychoeducation in a study assessing the effectiveness of CBT, or CBT in a study interested in MI).

#### Predictor or moderator

The potential treatment effect modifier we are interested in is defined as the level of motivation for change assessed at treatment entry through self-administered questionnaire, third-party administered questionnaire, or standardized interview. Studies looking at either the predictive or moderator effect of motivation will be included. Assessment tools based on different theories of motivation such as the Transtheoretical Stage of Change Model (TTM) or the Self-Determination Theory (SDT) will be included [24, 25]. Eligible motivation measurement instruments will include, but will not be limited to : the *Readiness and Motivation Interview for eating disorders* (RMI) [26], the *Anorexia Nervosa Stages of Change Questionnaire* (ANSOCQ) [27], the *Bulimia Nervosa Stages of Change Questionnaire* (BNSOCQ) [27], the *Eating Disorders Stages of Change Questionnaire* (EDSOCQ) [28], the *Readiness and Motivation Questionnaire* (RMQ) [29], the *Pros and Cons of Anorexia Nervosa* (P-CAN) [30], the *Pros and Cons of Eating Disorders Scale* (P-CED) [31], readiness rulers, the *University of Rhode Island Change Assessment Scale* (URICA) [32] and the *Autonomous and Controlled Motivations for Treatment Questionnaire* (ACMTQ) [33].

#### Outcomes of interest

End-of-treatment outcome will be measured in function of changed symptoms through self-administered questionnaire, third-party administered questionnaire or standardized interview administered at treatment entry and at the end of treatment. Eligible tools to measure changes in ED-related symptoms will include but will not be limited to the *Eating Disorder Examination* interview (EDE) [34, 35], the *Eating Disorder Examination Questionnaires* (EDE-Q1.0-6.0) [36], the *Eating Attitudes Tests* (EAT-26 and EAT-40) [37, 38] and the *Eating Disorder Inventories* (EDI, EDI-2, EDI-3) [39–41]. Validated instruments assessing for psychiatric comorbidities will also be admissible.

Studies including at least one of the following outcomes will be included:

1. Change in overall ED symptomatology as measured by a questionnaire’s total scores.
2. Change in ED related concerns including a) restraint, b) shape concerns, c) eating concerns, and d) weight concerns defined as the difference between post- and pre-treatment scores for each concern. They can be measured using the EDE-Q subscales or relevant subscales from other measurement tools;
3. Change in frequency of binging episodes;
4. Change in frequency of compensatory behaviours including a) vomiting, b) exercise intensity (this includes both exercise frequency and duration) and c) other compensatory behaviours (laxative or diuretic use, etc);
5. In participants with AN, change in weight throughout treatment. This includes absolute weight gain in kilograms, grams, pounds or ounces, increase in Body Mass Index (BMI) and weight gain measured in percentage of ideal body weight;
6. Change in the severity of psychiatric comorbidities such as depression, general anxiety or obsessive-compulsive disorders as measured with scales at beginning and end of therapy;
7. Drop-out rate from treatment.

#### Timing

Studies involving treatments of all lengths will be included.

#### Setting

Inpatient and outpatient studies will both be included given that published research has demonstrated that motivation for change has an impact on treatment outcomes even in patients receiving more intensive types of treatment [42].

No date limits or geographical restrictions will be set. Unpublished abstracts will not be excluded. Articles in languages other than English, French, or Spanish will be excluded.

### Search strategy

A systematic review will be conducted in the following databases: MEDLINE (Ovid MEDLINE(R) In-Process & Other Non-Indexed Citations, Ovid MEDLINE(R) Daily, Ovid MEDLINE(R) and Ovid OLDMEDLINE(R), 1946 to present), PsychINFO (Ovid, 1987 to present), Cochrane library (Wiley, current issue) and Embase (EMBASE Classic, OVID 1947-1973 (Ovid) and Embase current 1996- (Ovid)). The choice of databases was discussed with a university librarian. In addition, bibliographies of relevant articles will be screened for additional eligible studies and research registrars (ClinicalTrials.gov, ISRCTN registry and International Clinical Trials Registry Platform by the World Health Organisation) will be searched for relevant unpublished studies or abstracts. PROSPERO will also be searched for ongoing or recently published systematic reviews. Finally, experts in the field of EDs and motivation will be solicited and given a bibliography of the included studies with the objective of identifying missed or unpublished pertinent articles. The experts will be selected with the help of a senior clinician and researcher in the field of EDs, based on the prominence of their work on motivation in EDs.

Specific search strategies for each database were developed with the help of a university librarian specialized in psychiatry and knowledgeable about systematic review searching. Scoping searches to identify key articles in the field were first performed to retrieve appropriate keywords. A preliminary search strategy was then developed on MEDLINE (Ovid) using terms related to EDs, motivation, and outcomes with input from JS, HS, and MI. It was verified that the key articles previously found by the scoping searches were captured by the search strategy. Once the MEDLINE (Ovid) search strategy was finalized, the syntax and subject headings were adapted to the other databases. No limits or search filters will be applied. The searches were then reviewed by a second university librarian specialized in psychiatry. None of the librarians had interest in the project. The MEDLINE (Ovid) search strategy can be found in Table 1.

**Table 1:** MEDLINE search strategy

1. eating disorders/
2. anorexia nervosa/
3. bulimia nervosa/
4. anorexia/
5. bulimia/
6. Binge-Eating Disorder/
7. eating disorder*or disordered eating.mp.
8. anorexi*.mp.
9. bulimi*.mp.
10. 1 or 2 or 3 or 4 or 5 or 6 or 7 or 8 or 9
11. motivation/
12. readiness.mp.
13. motiv*.mp.
14. preparation for change.mp.
15. 11 or 12 or 13 or 14
16. treatment outcome/
17. treatment refusal/
18. patient dropout/
19. patient compliance/
20. weight gain/
21. outcome*.mp.
22. prognos*.mp.
23. (comply* or complied or compliant or complianc*).mp.
24. (dropout* or drop* out*).mp.
25. (refuse* or refusal* or refusing).mp.
26. (purging or purge* or binge* or binging or restrict* or compensatory behavi* or exercis* or laxative*).mp.
27. abstinen*.mp.
28. recover*.mp.
29. remission*.mp.
30. nutritional rehabilitation.mp.
31. weight gain*.mp.
32. weight restoration.mp.
33. treatment respons*.mp.
34. response* to treatment*.mp.
35. treatment adherence.mp.
36. adherence to treatment*.mp.
37. or/16–36
38. 10 and 15 and 37

### Data selection

All references retrieved by the search strategy will be uploaded to Eppi-Reviewer 4 software. This web-based software acts as a reference manager, allows for identification of duplicates, study classification, and data extraction using pre-created forms. It also facilitates production of diagrams such as the PRISMA flow diagram and allows statistical analysis. All reviewers will have access to the software online, which will simplify the study selection procedure.

Prior to the formal screening process, double references will be excluded and pilot testing of an inclusion/exclusion criteria form created on Eppi-Reviewer 4 will be done on 30 titles and abstracts by two independent reviewers to minimize later disagreement. An example of the inclusion/exclusion form can be found in Table 2. Screening will then be performed on the basis of titles and abstracts in an unblinded standardized manner by the same two independent reviewers using the research question and the inclusion/exclusion criteria form previously tested. Full texts of articles selected during this phase will then be uploaded to the software and will be assessed for eligibility by the same two unblinded independent reviewers using a similar inclusion/exclusion criteria form, previously piloted on 10 full records. We will log the reasons for excluding records. In case of unresolved discrepancies, a third party will be consulted at all phases. In order to minimize bias, we will pay particular attention to studies published by the same authors. We will try to identify reports that used the same sample of patients by looking at sample sizes, intervention and outcome descriptions, and years the studies were conducted. If we are confident that more than one report is issued from the same study, only one will be included. Inter-rater agreement will be calculated using kappa statistics at all steps.

**Table 2:** Screening on full text form

EXCLUDE on population diagnosis: EXCLUDE if participants are NOT receiving treatment for a diagnosis of anorexia nervosa, bulimia nervosa, binge-eating disorder or other specified feeding or eating disorder relying upon established criteria such as DSM or ICD.
EXCLUDE on absence of predictor or moderator: EXCLUDE if motivation for change is NOT being measured at treatment entry. Synonyms of motivation for change include: motivation for treatment, readiness for change and motivation for therapy.
EXCLUDE on study design: EXCLUDE books and book chapters or similar “information only” articles, guidelines, case series, case reports, qualitative studies, cohort studies, cross sectional studies, and reviews. Protocols and abstracts must be INCLUDED.
EXCLUDE on intervention length: EXCLUDE if treatment is shorter than 2 weeks or if self-help material seems too brief to take at least 2 weeks to cover at a rhythm of 1 h per week.
EXCLUDE on population age: EXCLUDE if participants are younger than 10 years old.
EXCLUDE on intervention type: EXCLUDE if the intervention is NOT psychotherapeutic (psychoeducation, CBT, FBT, DBT, MI, MET, CRT, etc.). App/web/manual-based self-help approaches can be included (see protocol for details). Other approaches such as medication, yoga or exercise will be excluded.
EXCLUDE on absence of outcome studied: EXCLUDE if the study does not present statistics concerning the impact of motivation for change (predictor or moderator) on outcomes studied. Outcomes are: Change in ED total symptomatology, change in shape concerns, change in weight concerns, change in eating concerns, change in restrictive behaviour, change in vomiting frequency, change in binging frequency, change in exercising behaviour, change in other purging behaviour (use of laxatives or diuretics, etc), change in psychiatric comorbidities, dropout rate, in AN, change in weight (absolute weight, BMI, fat %, Ideal body weight). Relapse rate and re-hospitalisation are EXCLUDED.
INCLUDE on full study.

### Data extraction

Data from included studies will be extracted using a standardized code set created using Eppi-Reviewer 4 software and inspired from the Cochrane Collaboration data extraction form template for intervention reviews [43]. The data extraction form will be pilot-tested on four eligible articles prior to starting the extraction process and will be improved accordingly. One author will extract the data and a second author will check for accuracy. Disagreements will be resolved by discussion and if no consensus arises, a third author will be asked to decide. No additional study will be included in the review once the data extraction process has started.

Data extracted will consist of:

1. Study characteristics: title of the study, authors’ names, year of publication, years the study was conducted, country, and language.
2. Study methodology: study design, trial setting, and theory of motivation. For both intervention and comparison arm, number and length of therapy sessions, type of therapy, way of administering therapy, total length of treatment and length of follow-up will be recorded. For each outcome, sample size at recruitment, sample size at completion of the study and completion rate as well as number of measurements and time-points will also be compiled. Likewise, the names of the motivation, ED severity, and comorbidity measurement tools will be documented, along with a description of the tools.
3. Participant characteristics: mean age, sex, type of ED, use of medication, duration of illness, number of prior treatment trials, number of prior hospitalisations, psychiatric comorbidities, ethnicity, and socioeconomic status.
4. Study results: Results for the outcomes outlined below and the statistical methods used will be logged. When studies report response to treatment at different points in time, all time points will be recorded.

We will take note of whether or not the information provided is sufficient to enable calculation of an effect size. Should needed statistics not be available, they will be approximated based on available inferential statistics or data presented in figures. If effect sizes cannot be calculated, study authors will be contacted for additional information on up to two occasions. Correspondence with authors will be tabulated.

### Outcomes of interest

The primary outcome will be the change in overall ED symptomatology between end and beginning of treatment, as measured with one of the tools aforementioned. We chose total symptom change as the primary outcome because we anticipate that this will be the outcome most frequently reported across studies.

Secondary outcomes will be defined as:

1. Change in ED-related concerns including a) restraint, b) shape concerns, c) eating concerns, and d) weight concerns defined as the difference between post- and pre-treatment scores for each concern;
2. Change in frequency of binging episodes;
3. Change in frequency of compensatory behaviours including a) vomiting, b) exercise intensity, and c) other compensatory behaviours (laxative or diuretic use, etc);
4. In participants with AN, change in weight throughout treatment;
5. Change in the severity of psychiatric comorbidities such as depression, anxiety, or obsessive-compulsive disorder as measured with scales at beginning and end of therapy;
6. Drop-out rate.

### Quality assessment

A 20-item risk of bias assessment form was derived from a combination and an adaptation of 3 high quality tools in order to accurately assess all study designs included in the present review [44–46]. It assesses randomization (allocation bias), comparability of the groups (confounding factors), eligibility criteria (selection bias), blinding (detection bias), questionnaire validity (measurement bias), withdrawal (attrition bias) and outcomes (reporting bias and outcome misclassification). Reviewers’ response options will be limited to yes, no, partially, not stated, or not applicable.

The checklist will be created on Eppi-Reviewer 4. It will first be pilot tested on one article by 2 reviewers to make sure that they both use the tool the same way. The same 2 raters will then independently assess the quality of all included articles in duplicate. Disagreements will be resolved by discussion and a third party will be consulted for arbitration if disagreement persists. Inter-rater agreement will be calculated with kappa statistic. Reviewers will not be blinded to studies and quality assessment will be conducted following data extraction.

Quality assessment results will be represented in a table. No attempt to give an overall quality score to the studies will be made. Poor quality studies will not be excluded from data synthesis but a sensitivity analysis will be performed.

### Data synthesis

For each outcome and each time-point available in original studies, an effect size will be calculated if provided information permits. Every type of ED concern and compensatory behaviour will be treated as a separate outcome. When two scales report on the same outcome in a study (e.g. both EDE-Q and EAT total scores are reported), one combined effect size will be computed accounting for the two measures. Effect sizes will be expressed as a correlation coefficient *r*. Correlation coefficients will be computed by logging individual study results in a publicly available effect size calculator [47]. We chose this metric as an initial description of findings since it is familiar to most readers. Separate meta-analyses will be executed for each outcome for which findings from 3 or more trials can be pooled. No attempt will be made to pool all outcomes into one meta-analysis given that previous research indicates a different magnitude of effect of motivation on each symptom studied [16]. Aggregate results will be statistically combined using weighted averages calculated using a random-effects model. The Hedges and Vevea method of meta-analysis will be used, [48] in which effect sizes are first converted into a standard *z* metric before calculating the average of the scores.

When studies report response to treatment at different points in time, the time points given in original studies as the treatment endpoint will be statistically combined, as well as a time point at follow-up between 2 and 6 months, and a time-point at over 6 months after treatment end. When more than one measure of treatment outcome is recorded between 2 and 6 months after treatment completion, the time point closest to the 6 months’ endpoint will be given preference. When more than one measure of treatment outcome is recorded more than 6 months after treatment completion, the time-point closest to the 1 year endpoint will be selected.

Since the eating-disordered population is characterized by relatively high dropout rates, several studies do not adopt intent-to-treat analysis. Intent-to-treat analyses risk in this case to produce overly conservative results that would mask true associations. Accordingly, we will favour raw statistics rather than intent-to-treat statistics in our analyses. Studies of all sizes and all qualities will be included in the analyses.

A forest plot of the included studies will be created and a first assessment of heterogeneity will be made by visual perusal. Statistical heterogeneity will also be evaluated using Cochran’s Q statistic. A p-value of 0.1 will determine significance. I^2^ statistic will subsequently be calculated to measure the degree of heterogeneity (0–25% negligible heterogeneity, 25–50% low heterogeneity, 50–75% moderate heterogeneity, 75–100% high heterogeneity).

If significant heterogeneity is observed, we will explore clinical and methodological correlates of heterogeneity further by performing subgroup and sensitivity analyses on the following variables:

1. Diagnosis (AN, BN, BED, and OSFED)
2. Age group (Adolescents versus adults)
3. Treatment setting (Inpatient versus outpatient)
4. Motivation theory (SDT versus TTM)
5. Study quality (excluding low quality studies, excluding outlier studies identified by eyeballing)
6. Study design (RCT versus others, if RCTs are retrieved)

A systematic narrative synthesis will be provided in addition to the meta-analysis. It will include a flow diagram, tables on study characteristics, patient characteristics, study results, quality assessment, and a forest plot. Text will also summarize relevant relationships between and within included studies.

### Assessment of meta-biases

Funnel plots of trials’ standardized mean differences will be used in order to investigate the possibility of publication bias if 10 studies or more are available. It will be assessed visually for small study effects.

To gauge the likelihood of selective reporting bias, we will look at published protocols of included studies if they exist and compare outcomes reported in the protocol with those described in the published review. We will search for published protocols on ClinicalTrials.gov, ISRCTN registry, and International Clinical Trials Registry Platform by the World Health Organisation. If no protocol is available, we will compare the outcomes listed in the methods sections of included reviews with those for which results are presented in the articles and search for omissions.

### Dissemination of results

Results of this systematic review will be disseminated by presentations at national and international conferences and by publication of one or more peer-reviewed articles in ED related journals.

## Discussion

The strengths of this systematic review include: a transparent, comprehensive, and systematic methodology; a search strategy that will be openly accessible and broad in terms both of wording and database searches; an exhaustive formal quality assessment that will be undertaken to evaluate risk of biases within and across included studies bias; sensitivity analyses that will be performed to account for heterogeneity in clinical and methodological characteristics across trials.

The challenges of undertaking this systematic review will lie in the limitations of existing literature and the clinical and methodological heterogeneities observed across included papers: it is expected that the literature found on the topic will be abundant, although of limited quality; most of the studies will be before-after studies rather than RCTs which are less prone to bias introduction; study samples will frequently be small and subject to high attrition; type and length of treatments will vary, and so will reported outcomes; both motivation and outcomes will be measured through questions to participants and are thus subject to their subjectivity; various questionnaires will be used to assess motivation as there is no consensus on the tool to be used [49]. For all the above reasons, it is probable that heterogeneity will be found across trials.

Nonetheless, this systematic review will be the first high quality attempt to synthesize the literature on the impact of motivation at treatment entry on outcome in EDs. It will determine and quantify the predictive value of level of motivation at treatment entry on treatment outcomes in EDs. Identifying modifiable predictors of good and poor outcomes in EDs is critical in order to improve treatment efficacy and develop innovative treatment approaches.

## Additional file

Additional file 1: Prisma-P checklist. Prisma-P checklist. (DOCX 32 kb)

## Abbreviations

ACMTQ: Autonomous and Controlled Motivations for Treatment Questionnaire
AN: Anorexia nervosa
ANSOCQ: Anorexia Nervosa Stages of Change Questionnaire
BED: Binge-eating disorder
BMI: Body mass index
BN: Bulimia nervosa
BNSOCQ: Bulimia Nervosa Stages of Change Questionnaire
CBT: Cognitive behavioural therapy
CCT: Clinical controlled trial
CI: Confidence Interval
DBT: Dialectic behavioural therapy
DSM: Diagnostic and Statistical Manual of Mental Disorders
EAT: Eating attitudes tests
ED: Eating disorder
EDE: Eating disorder examination interview
EDE-Q: Eating disorder examination questionnaires
EDI: Eating disorder inventories
EDSOCQ: Eating Disorders Stages of Change Questionnaire
FBT: Family based therapy
ICD: International Classification of Diseases
ISRCTN: International Standard Randomised Controlled Trials Number
MEDLINE: Medical Literature Analysis and Retrieval System Online
MI: Motivational Interviewing
P-CAN: Pros and Cons of Anorexia Nervosa
P-CED: Pros and Cons of Eating Disorders Scale
PRISMA: Preferred Reporting Items for Systematic Reviews and Meta-Analyses
PROSPERO: Prospective Register of Systematic Reviews
PsychINFO: Database of Psychological Literature
RCT: Randomized controlled trial
RMI: Readiness and Motivation Interview for eating disorders
RMQ: Readiness and Motivation Questionnaire
SDT: Self-Determination Theory
TTM: Transtheoretical Stage of Change Model
URICA: University of Rhode Island Change Assessment Scale
